# Supplementary material for: Interactions between chaperone and energy storage networks during the evolution of Legionella pneumophila under heat shock
Source: PeerJ. 2024 Apr 30;12:e17197. doi: 10.7717/peerj.17197 (PMC11067923; doi:10.7717/peerj.17197)
Supplement: Supplemental Information 2 [file peerj-12-17197-s002.docx]

**Supplemental Table S1: Primers used in this manuscript**

| **Primer** | **Sequence (5’-3’)** | **Source** |
| --- | --- | --- |
| phaP-UF | ACTCTTGGGCTTAAAGAAGCA | This study |
| phaP-UR | CAGTCTAGCTATCGCCATGTATATCTAGCAGTTGGCGGCTG | This study |
| phaP-DF | GATGCTGAAGATCAGTTGGGTATACCCAAGCGAGTCAATTAA | This study |
| phaP-DR | TCCCAATGCTCGTTATGACC | This study |
| phaP-KnF | CAGCCGCCAACTGCTAGATATACATGGCGATAGCTAGACTG | This study |
| phaP-KnR | TTAATTGACTCGCTTGGGTATACCCAACTGATCTTCAGCATC | This study |
| htpG-UF | GTCGCTGGATCCCTGAGTTA | This study |
| htpG-UR | CAGTCTAGCTATCGCCATGTATTGCTAATGCTTGAGACAATATTCA | This study |
| htpG-DF | GATGCTGAAGATCAGTTGGGTGCAATATGAATAATCACCAATAGAT | This study |
| htpG-DR | TCCATGTTTGGCTTCATGCTG | This study |
| htpG-KnF | TGAATATTGTCTCAAGCATTAGCAATACATGGCGATAGCTAGACTG | This study |
| htpG-KnR | ATCTATTGGTGATTATTCATATTGCACCCAACTGATCTTCAGCATC | This study |
| clpX-UF | TGCTTCAGCAGACATCGGAG | This study |
| clpX-UR | GATGCTGAAGATCAGTTGGGTCGAATTGTTCACCAGCACCAAT | This study |
| clpX-DF | CAGTCTAGCTATCGCCATGTATAATGTGAGAGTTAGTCTATTGC | This study |
| clpX-DR | GATAGTAATGTGGTGGCGCA | This study |
| clpX-KnF | GCAATAGACTAACTCTCACATTATACATGGCGATAGCTAGACTG | This study |
| clpX-KnR | ATTGGTGCTGGTGAACAATTCGACCCAACTGATCTTCAGCATC | This study |
| mreC-UF | CAAAGGCACACGTGATGGTG | This study |
| mreC-UR | GGCGATTCAGGTTCATCATGCACTCCAGCCCGGGGAATATAA | This study |
| mreC-DF | CGGTAAATTGTCACAACGCCGTGATCTAAGTGCCAATCACCA | This study |
| mreC-DR | CGGTAAAAATGCGGTTGGGA | This study |
| mreC-GnF | TTATATTCCCCGGGCTGGAGTGCATGATGAACCTGAATCGCC | This study |
| mreC-GnR | CCAGCGTAAAATAGGCAATCCACGGCGTTGTGACAATTTACCG | This study |
| dnaJ-UF | CCTCATCTACAGCAAAACCATGA | This study |
| dnaJ-UR | CGGTAAATTGTCACAACGCCGTGGATTGCCTATTTTACGCTGG | This study |
| dnaJ-DF | GGCGATTCAGGTTCATCATGCATGCTGAAAACGACAAATCTTCC | This study |
| dnaJ-DR | TAAATTTTGGGTCGGGGCGA | This study |
| dnaJ-GnF | GGAAGATTTGTCGTTTTCAGCATGCATGATGAACCTGAATCGCC | This study |
| dnaJ-GnR | TGGTGATTGGCACTTAGATCACGGCGTTGTGACAATTTACCG | This study |
| rodA-UF | GGCATGAAACCTGCCAAGAT | This study |
| rodA-UR | GCAGCGAATGCCGGGTAATAACTTTGAAATCAAATCCGCAACA | This study |
| rodA-DF | CGTTCTTCGGGGCGAAAACAAAACTCTGAGTGGTATTAATTTGG | This study |
| rodA-DR | CGCTTCCACTGTAGCACGTA | This study |
| rodA-CmF | CCAAATTAATACCACTCAGAGTTTTGTTTTCGCCCCGAAGAACG | This study |
| rodA-CmR | TGTTGCGGATTTGATTTCAAAGTTATTACCCGGCATTCGCTGC | This study |
| clpB-UF | ACTCATTGGCCAGAATGAAGC | This study |
| clpB-UR | CGTTCTTCGGGGCGAAAACGGTCTTGAAACATTGCCCCG | This study |
| clpB-DF | GCAGCGAATGCCGGGTAATAAAGCTGGGTAAACATATCAAAC | This study |
| clpB-DR | GAGCTTTGCTTCATCGCTATT | This study |
| clpB-CmF | CGGGGCAATGTTTCAAGACCGTTTTCGCCCCGAAGAACG | This study |
| clpB-CmR | GTTTGATATGTTTACCCAGCTTTATTACCCGGCATTCGCTGC | This study |
| dnaK-UF | GAATCCTTCTTCCGCCCTCT | (Liang et al., 2023) |
| dnaK-UR | GCAGCGAATGCCGGGTAATAAATCGTCATGATGCACCCCAT | (Liang et al., 2023) |
| dnaK-DF | CGTTCTTCGGGGCGAAAACTCAAGCTATCACCCTAAAAAAAGC | (Liang et al., 2023) |
| dnaK-DR | ATGGACAATTACTGCAAGCCA | (Liang et al., 2023) |
| dnaK-CmF | GCTTTTTTTAGGGTGATAGCTTGAGTTTTCGCCCCGAAGAACG | (Liang et al., 2023) |
| dnaK-CmR | ATGGGGTGCATCATGACGATTTATTACCCGGCATTCGCTGC | (Liang et al., 2023) |
