# Supplementary material for: Interactions between chaperone and energy storage networks during the evolution of Legionella pneumophila under heat shock
Source: PeerJ. 2024 Apr 30;12:e17197. doi: 10.7717/peerj.17197 (PMC11067923; doi:10.7717/peerj.17197)
Supplement: Supplemental Information 3 [file peerj-12-17197-s003.docx]

| **Primer** | **Sequence (5’-3’)** | **Amplicon Length (bp)** | **Efficiency** | ***r^2^*** |
| --- | --- | --- | --- | --- |
| dnaK-qPCR-F | CGCAATAACAGAGTCACCGC | 116 | 90.9% | 0.999 |
| dnaK-qPCR-R | GCAACCAAAGATGCTGGTCG |  |  |  |
| dnaJ-qPCR-F | TTTGTCCACGTGATTGACGC | 125 | 92.7% | 0.999 |
| dnaJ-qPCR-R | ATCCTTCAATGGGTGGAGGC |  |  |  |
| rpoH-qPCR-F | CCCATCTTGGGGTCAAAACG | 78 | 94.1% | 1.000 |
| rpoH-qPCR-R | CGCGGTTATCTGGGGTATGG |  |  |  |
| 16S-qPCR-F | AGAGATGCATTAGTGCCTTCGGGA | 113 | 95.0% | 0.999 |
| 16S-qPCR-R | ACTAAGGATAAGGGTTGCGCTCGT |  |  |  |
