# Supplementary material for: Interactions between chaperone and energy storage networks during the evolution of Legionella pneumophila under heat shock
Source: PeerJ. 2024 Apr 30;12:e17197. doi: 10.7717/peerj.17197 (PMC11067923; doi:10.7717/peerj.17197)
Supplement: Supplemental Information 8 [file peerj-12-17197-s008.docx]

**Supplemental Table S7: Bacterial strains and plasmids used in this manuscript**

| **Strain** | **Description** | **Strain Bank ID** | **Source** |
| --- | --- | --- | --- |
| Philadelphia-1 | Philadelphia clinical isolate from 1976 outbreak: ATCC33152 |  | American Type Culture Collection |
| KS79 | JR32 ∆comR |  | (De Felipe et al., 2008) |
| HA-1.(5, 10, etc.) | Populations of heat-adapted *L. pneumophila* from the **first** replicate lineage sampled at five-passage intervals |  | This study |
| HA-2.(5, 10, etc.) | Populations of heat-adapted *L. pneumophila* from the **second** replicate lineage sampled at five-passage intervals |  | This study |
| HA-3.(5, 10, etc.) | Populations of heat-adapted *L. pneumophila* from the **third** replicate lineage sampled at five-passage intervals |  | This study |
| TRA-1.1 | KS79 *dnaK*::*dnaK*^M94I^ - CHL^pMMB207c^ | SPF714 | (Liang et al., 2023) |
| TRA-1.2 | KS79 *dnaK*::*dnaK*^M94I^ - CHL^pMMB207c^, *dnaJ*::*dnaJ*^F95Y^ - GEN^pBBR1-MCS2^ | SPF697 | This study |
| TRA-1.3 | KS79 *dnaK*::*dnaK*^M94I^ - CHL^pMMB207c^, *dnaJ*::*dnaJ*^F95Y^ - GEN^pBBR1-MCS2^, *clpX*::*clpX*^G202A^ - KAN^pSF6^ | SPF699 | This study |
| TRA-2.1 | KS79 *phaP*::*phaP*^Δ1bp^ - KAN^pSF6^ | SPF605 | This study |
| TRA-2.2 | KS79 *phaP*::*phaP*^Δ1bp^ - KAN^pSF6^, *mreC*::*mreC*^V266A^ - GEN^pBBR1-MCS2^ | SPF686 | This study |
| TRA-2.3 | KS79 *phaP*::*phaP*^Δ1bp^ - KAN^pSF6^, *mreC*::*mreC*^V266A^ - GEN^pBBR1-MCS2^, *rodA*::*rodA*^R112S^ - CHL^pMMB207c^ | SPF713 | This study |
| TRA-3.1 | KS79 *dnaK*::*dnaK*^V373L, M94I^ - CHL^pMMB207c^ | SPF715 | (Liang et al., 2023) |
| TRA-3.2 | KS79 *dnaK*::*dnaK*^V373L, M94I^ - CHL^pMMB207c^, *dnaJ*::*dnaJ*^F95Y^ - GEN^pBBR1-MCS2^ | SPF696 | This study |
| TRA-3.3 | KS79 *dnaK*::*dnaK*^V373L, M94I^ - CHL^pMMB207c^, *dnaJ*::*dnaJ*^F95Y^ - GEN^pBBR1-MCS2^, *clpX*::*clpX*^E203D^ - KAN^pMMB207c^ | SPF703 | This study |
| TRA-4.1 | KS79 *clpB*::*clpB*^A478V^ - CHL^pMMB207c^ | SPF707 | This study |
| TRA-4.2 | KS79 *dnaJ*::*dnaJ*^P334S^ - GEN^pBBR1-MCS2^ | SPF609 | This study |
| TRA-4.3 | KS79 *htpG*::*htpG*^G83E^ - KAN^pSF6^ | SPF607 | This study |
| TRA-4.4 | KS79 *clpB*::*clpB*^A478V^ - CHL^pMMB207c^, *dnaJ*::*dnaJ*^P334S^ - GEN^pBBR1-MCS2^ | SPF708 | This study |
| TRA-4.5 | KS79 *clpB*::*clpB*^A478V^ - CHL^pMMB207c^, *htpG*::*htpG*^G83E^ - KAN^pSF6^ | SPF718 | This study |
| TRA-4.6 | KS79 *dnaJ*::*dnaJ*^P334S^ - GEN^pBBR1-MCS2^, *htpG*::*htpG*^G83E^ - KAN^pSF6^ | SPF692 | This study |
| TRA-4.7 | KS79 *clpB*::*clpB*^A478V^ - CHL^pMMB207c^, *dnaJ*::*dnaJ*^P334S^ - GEN^pBBR1-MCS2^, *htpG*::*htpG*^G83E^ - KAN^pSF6^ | SPF709 | This study |
| PJ | KS79 *phaP*::*phaP*^Δ1bp^ - KAN^pSF6^, *dnaJ*::*dnaJ*^F95Y^ - GEN^pBBR1-MCS2^ | SPF732 | This study |
| PK1 | KS79 *phaP*::*phaP*^Δ1bp^ - KAN^pSF6^, *dnaK*::*dnaK*^M94I^ - CHL^pMMB207c^ | SPF730 | This study |
| PK1J | KS79 *phaP*::*phaP*^Δ1bp^ - KAN^pSF6^, *dnaJ*::*dnaJ*^F95Y^ - GEN^pBBR1-MCS2^, *dnaK*::*dnaK*^M94I^ - CHL^pMMB207c^ | SPF734 | This study |
| PK2 | KS79 *phaP*::*phaP*^Δ1bp^ - KAN^pSF6^, *dnaK*::*dnaK*^V373L, M94I^ - CHL^pMMB207c^ | SPF738 | This study |
| PK2J | KS79 *phaP*::*phaP*^Δ1bp^ - KAN^pSF6^, *dnaJ*::*dnaJ*^F95Y^ - GEN^pBBR1-MCS2^, *dnaK*::*dnaK*^V373L, M94I^ - CHL^pMMB207c^ | SPF739 | This study |
| **Plasmid** | **Description** |  | **Source** |
| pMMB207c | RSF1010 derivative, IncQ, lacIq, CHL^r^, Ptac, oriT, ∆mobA |  | (Chen et al., 2004) |
| pSF6 | pGEMT-easy-rrnb, KAN^r^ |  | (Faucher et al., 2011) |
| pBBR1-MCS2 | GEN^r^ |  | (Kovach et al., 1995) |
